# Effect of *Spirulina* Dietary Supplementation in Modifying the Rumen Microbiota of Ewes

**DOI:** 10.3390/ani13040740

**Published:** 2023-02-19

**Authors:** Christos Christodoulou, Alexandros Mavrommatis, Dimitris Loukovitis, George Symeon, Vassilios Dotas, Basiliki Kotsampasi, Eleni Tsiplakou

**Affiliations:** 1 Laboratory of Nutritional Physiology and Feeding, Department of Animal Science, School of Animal Biosciences, Agricultural University of Athens, Iera Odos 75, 11855 Athens, Greece; 2 Department of Fisheries and Aquaculture, School of Agricultural Sciences, University of Patras, 30200 Messolonghi, Greece; 3 Research Institute of Animal Science, ELGO DIMITRA, 58100 Giannitsa, Greece; 4 Department of Animal Production, Faculty of Agriculture, Aristotle University of Thessaloniki, 54124 Thessaloniki, Greece

**Keywords:** ewes, microalgae, *Spirulina*, rumen, microorganisms

## Abstract

**Simple Summary:**

The supplementation of microalgae as additives in animal diets has gathered the scientific community’s attention over recent years due to their beneficial properties. A better understanding of the effect of such additives on the rumen physiology could unravel their mode of action and overall functionality. Therefore, this study investigated the effect of *Spirulina* supplementation on modifying the rumen microbiota of ewes. Our results suggested that supplementing *Spirulina*, in the highest studied level (15 g/ewe/day), resulted in significant alterations in the relative abundance of the rumen microorganisms, with those reported regarding the cellulolytic bacterial species being considered the most interesting.

**Abstract:**

Supplementing ruminant diets with microalgae, may prove an effective nutritional strategy to manipulate rumen microbiota. Forty-eight ewes were divided into four homogenous groups (*n* = 12) according to their fat-corrected milk yield (6%), body weight, age, and days in milk, and were fed individually with concentrate, alfalfa hay, and wheat straw. The concentrate of the control group (CON) had no *Spirulina* supplementation, while in the treated groups 5 (SP5), 10 (SP10), and 15 g (SP15) of *Spirulina* were supplemented as an additive in the concentrate. An initial screening using metagenomic next-generation sequencing technology was followed by RT-qPCR analysis for the targeting of specific microbes, which unveiled the main alterations of the rumen microbiota under the *Spirulina* supplementation levels. The relative abundance of *Eubacterium ruminantium* and *Fibrobacter succinogenes* in rumen fluid, as well as *Ruminococcus albus* in rumen solid fraction, were significantly increased in the SP15 group. Furthermore, the relative abundance of *Prevotella brevis* was significantly increased in the rumen fluid of the SP5 and SP10 groups. In contrast, the relative abundance of *Ruminobacter amylophilus* was significantly decreased in the rumen fluid of the SP10 compared to the CON group, while in the solid fraction it was significantly decreased in the SP groups. Moreover, the relative abundance of *Selenomonas ruminantium* was significantly decreased in the SP5 and SP15 groups, while the relative abundance of *Streptococcus bovis* was significantly decreased in the SP groups. Consequently, supplementing 15 g *Spirulina*/ewe/day increased the relative abundance of key cellulolytic species in the rumen, while amylolytic species were reduced only in the solid fraction.

## 1. Introduction

Microalgae can be considered an eco-efficient and sustainable source of animal feed because they are rich in bioactive compounds gathering the attention of animal nutritionists over recent decades [1]. The bioactive compounds present in microalgae may trigger antioxidant [2,3,4,5,6] and immunomodulatory properties [7,8]. Besides, they can beneficially enhance dairy product quality and improve their shelf life [2,3,4,5,6]. However, due to microalgae’s cell walls, their digestive efficiency remains an open debate. Moreover, microalgae exert antimicrobial activity [9], thus, evaluating the effect on the rumen microbiome may unveil important findings and help their holistic evaluation as animal feedstuff. Notably, the impact of microalgae in manipulating rumen microbiota has been previously demonstrated [10,11,12,13,14,15]. However, most of these studies attributed the alterations to the rumen microbiota to their ether extract and fatty acid content, and more specifically the mode of action of the presented polyunsaturated fatty acids (PUFA) such as docosahexaenoic acid (DHA). Although the nutritional benefits of microalgae in livestock have been well-described, the chaotic diversity of marine microbes, seaweeds, macro-, and micro-algae can variously affect animals’ metabolism. Beyond the interspecies differences in the chemical composition of algae, within species, the cultivation conditions can severely affect their composition as well [16,17]. Therefore, the effect of algae on animal nutrition should not be assessed by treating them as a uniform group, rather the focus needs to be on specific species while also considering their chemical composition.

*Spirulina*, the most cultivated microalga [18], is considered one of the most promising ones, and has been considered the “Food of the future”. It is an eco-friendly and sustainable microalga with antiviral, antioxidant, immunomodulating, and microbial-modulating activity [19]. Up until now, the use of low-fat *Spirulina* in animal diets has investigated mainly the effect on animal productivity and its antioxidant and immunomodulatory properties. More specifically, in our previous study [6], the inclusion of 15 g *Spirulina*/ewe/day increased milk PUFA and ω-3 content while also improved its antioxidant capacity indicating promising insights for a prolong milk and dairy product shelf-life. Additionally, *Spirulina*-fed ewes had higher blood antioxidant enzyme activities with lower values of protein carbonyls [6].

Nevertheless, it is essential to approach such a nutritional strategy holistically because the rumen microbiome is responsible for fermentation processes enabling the digestion of plant material and consequently is closely associated with ruminant productivity, product quality [20], and overall animal health. Moreover, the optimum microalgae supplementation level should also be assessed, as significant alterations in the rumen bacteriome have been associated with it in a dose-dependent manner [14]. Presumably, as there is no knowledge regarding the effect of the different levels of *Spirulina* supplementation, studying different levels could unravel different responses in manipulating rumen microbiota, enabling the evaluation of the optimum supplementation level.

Several molecular techniques were optimized to study the rumen microbiota and unravel novel features. An initial screening of the core bacteriome using a Next Generation Sequencing (NGS) technique, followed by targeting key microbe species with real-time qPCR (RT-qPCR), unveiled important findings regarding the effect of microalgae supplementation in goat and ewe diets [12,21]. Considering the above, this is the first study that has investigated the effect of three different supplementation levels of *Spirulina* on the rumen microbiota of ewes and key microbe species related to rumen fermentation processes using both NGS and RT-qPCR methods.

## 2. Materials and Methods

### 2.1. Experimental Diets and Experimental Design

Animal handling, housing, and care complied with the approved protocols by the Ethical Committee in Research of the Agricultural University of Athens. Forty-eight dairy Chios breed ewes were divided into four homogenous groups (*n* = 12) according to their fat corrected (6%) milk yield (FCM_6%_) (1.85 ± 0.3 kg/d), days in milk (67 ± 8 days), age (2–4 years old), and body weight (54 ± 6.0 kg). The feed ration consisted of alfalfa hay, wheat straw, and concentrate. The forages were provided separately from the concentrate as usually happens in traditional feeding systems for small ruminants. A detailed description of the housing and feeding system (individual feeding) is provided in our previously published study [6]. The composition of the four concentrates and the chemical composition of the feedstuffs are provided in Tables S1 and S2. Ewes were offered concentrate with the addition of three different levels of *Spirulina*. *Spirulina* was added manually as top dressing on the concentrate fraction of the diet. More specifically, the concentrate of the control group (CON) had no *Spirulina* supplementation, while in the three following dietary treatment groups (SP5, SP10, and SP15), *Spirulina* was supplemented as an additive at 5, 10, and 15 g/ewe/day, respectively. *Spirulina* was provided as a commercial organic product by Spirulina Nigrita Ltd. (Serres, Greece). Biomass was harvested with filtration and controlled solar drying was applied to ensure the ingredients integrity. The assays regarding the analysis of the collected feed samples are presented by Christodoulou et al. [6]. The average daily feed intake (g/ewe/day) and nutrient intake (g/ewe/day) of the four dietary treatments are presented in Table S3. Ewes had free access to fresh water.

### 2.2. Sample Collection

Rumen samples were collected on the final day of the experiment (60th experimental day). Rumen digesta samples were collected from each ewe before feeding using a stomach tube (flexible PVG tube of 1.5 mm thickness and 10 mm I.D.), and a vacuum pump (MZ2CNT, Vacuubrand Gmbh & Co Kg, Wertheim, Germany). The stomach tube was inserted at approximately 130 cm depth and during the collection, the tube was moved aiming to sample from different rumen places to avoid previously reported biases [22]. Up to 30 mL of rumen fluid was initially discarded to avoid saliva contamination [23]. To separate the solid fraction from the rumen fluid, four layers of cheesecloth were used. Following the sample collection, samples were snap-frozen in liquid nitrogen and then stored at −80 °C before lab analysis.

### 2.3. DNA Extraction

The DNA extraction was performed following the protocol described by Mavrommatis et al. [12]. To clear the RNA contamination, RNase A (10 mg/mL) was then added to each sample, followed by incubation at 37 °C for 1 h. Finally, the DNA pellet was resuspended in ultrapure water and purified through a NucleoSpin^®^ Tissue spin column (Macherey-Nagel GmbH & Co., KG, Düren, Germany) based on the manufacturer’s guidelines. From each sample, the quality of the extracted DNA was tested based on the abundance of the DNA content and the levels of impurities in the 260/230 and 260/280 ratios, using an ND-1000 spectrophotometer (Nanodrop, Wilmington, DE, USA), and verified in a 0.7% agarose gel.

### 2.4. Primer Design and Relative Abundance of Selected Rumen Microorganisms

The primer set that was considered for the real-time PCR, the genomic region of PCR amplification, the primer efficiency, amplicon size, and the hybridization temperature are provided in Table S4. Primer design, selection, amplification region, and validation processes were assessed following the steps described by Mavrommatis et al. [12]. A Step-One Plus Real-Time PCR System (Applied Biosystems, Foster City, CA, USA) was used for quantitative PCRs [12]. Primer specificity and primer dimer formation were investigated (melt curve) by applying dissociation curve analysis. Based on the equation provided by Carberry et al. [24]: relative abundance = e (target)^(Ct target microorganism-Ct of bacterial 16s rDNA)^, the proportion of total bacterial 16S ribosomal DNA was used to indicate the relative abundance of microbial populations. The relative abundance expression of the results is a practical and consistent method [25], given the fact that comparisons across treatments are limited and no other taxa are compared.

### 2.5. Metagenomic NGS Analysis

The DNA samples, (50 ng·uL) from each rumen fluid sample, were pooled for each group (four samples were obtained) for the metagenomic NGS bacteriome screening. The 16S rRNA gene (~1.5 kb) was amplified using a 16S Barcoding kit (SQK-RAB204, Oxford Nanopore Technologies (ONT), Oxford, UK), and following the manufacturer’s protocol. The sequencing library for 16S rRNA gene sequencing was generated from 20 ng of DNA using the same kit (SQK-RAB204 from Oxford Nanopore Technologies, Oxford, UK) following the manufacturer’s instructions and loaded into a MinION flow cell (R9.4.1, FLO-MIN106). The flow cell was placed into a MinION-Mk1B device (Oxford Nanopore Technologies) for sequencing and controlled using ONT’s MinKNOW software. Raw sequencing data (FAST5 files) were basecalled with algorithms implemented in GUPPY software (ONT) and reads were demultiplexed according to the used barcodes. Clean sequences were obtained after trimming of barcodes, adapter, and primer sequences. Processed reads (FASTQ files) were uploaded to the EPI2ME cloud-based workflow (Metrichor, Oxford, UK) for taxonomic classification of bacteria in the following major ranks: Superkingdom/phylum/class/order/family/genus/species. The analysis was carried out using NCBI’s ‘16S Ribosomal RNA database’ (bacterial and archaeal strains) with an identity threshold of 85% and a minimum quality score of 12.

### 2.6. Statistical Analysis

Statistical analysis was carried out using IBM SPSS Statistics for Windows software (IBM Corp. Released 2016. IBM SPSS Statistics for Windows, Version 24.0. Armonk, NY, USA). To compare the effect of the dietary treatment the one-way ANOVA analysis was performed (CON vs. SP5 vs. SP10 vs. SP15) on the relative abundance of the studied microorganisms.

The Lavene’s test was used to evaluate the homogeneity of the dataset and the Shapiro–Wilk test was used to assess the dataset’s normality. For the data that did not violate the homogeneity and normality tests, post hoc analysis was assessed considering the Tukey multiple range tests, while for the data that violated these criteria, considering the Games–Howell test. The significance threshold for these tests was set at 0.05. GraphPad Prism 8.4.2. software was used for the interleaved bars on which the error bars represent the standard error mean (SEM).

## 3. Results

### 3.1. 16S Amplicon Sequencing

Metagenomic sequencing of the DNA of the isolated microorganisms from the pooled rumen fluid samples resulted in the detection of 19, 20, 17, and 19 phyla, 50, 48, 53, and 49 families, and 108, 90, 91, and 90 genera for the CON, SP5, SP10, and SP15 groups, respectively. A minimum similarity criterion of 85% was set, and approximately 100,000 reads/sample were assigned to various taxonomic levels with an average identity score of 89% and an average quality score of 12.36. All the samples reached the plateau phase, consistent with the rarefaction curves of the bacterial population at the genus taxonomic level so that any additional increase in the number of sequences would not affect the number of genera discovered. The dominant rumen fluid phyla were Bacteroidetes (%), Firmicutes (%), and Proteobacteria (%). The highest relative abundance of the Bacteroidetes was observed in the CON (39.3%) compared to the other groups (SP5: 26.5%; SP10: 36.1%; and SP15: 29.2%, respectively). The relative abundance of Firmicutes was found to be higher in the SP15 group (63.0%) compared to the rest (CON: 50.7%; SP5: 59.4%; and SP10: 55.3%, respectively). The highest relative abundance of the Proteobacteria, was found in the SP5 (12.5%) compared to the rest (CON: 7.9%; SP10: 6.2%; and SP15: 5.8%, respectively). The Firmicutes:Bacteroidetes ratio was found to be at the lowest level for the CON group compared to the rest (Table 1).

**Figure 1:**
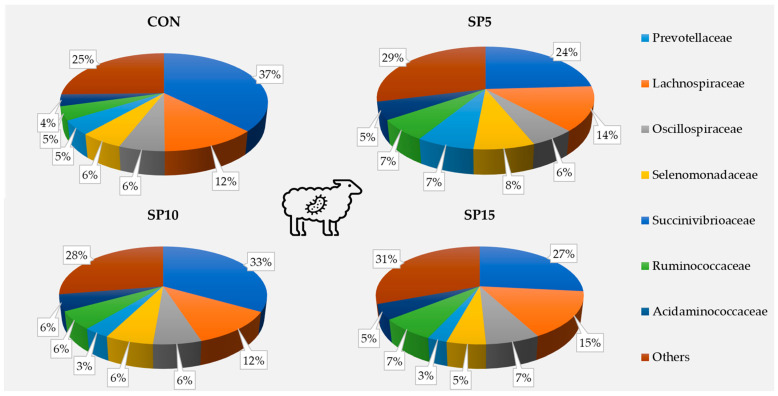
Relative abundance of dominant families based on the 16S amplicon sequencing in the rumen fluid (*n* = 1; pooled group samples) of the four pooled DNA samples representative of the four dietary treatments (CON; SP5; SP10; and SP15) with four different *Spirulina* supplementation levels (0, 5, 10, and 15 g/day, in the concentrate, respectively) at a family level.

At a family level, Prevotellaceae was dominant in the four dietary treatments (CON: 37.4%; SP5: 24.1%; SP10: 32.8%; SP15: 26.6%, respectively), followed by Lachnospiraceae (Figure 1).

**Figure 2:**
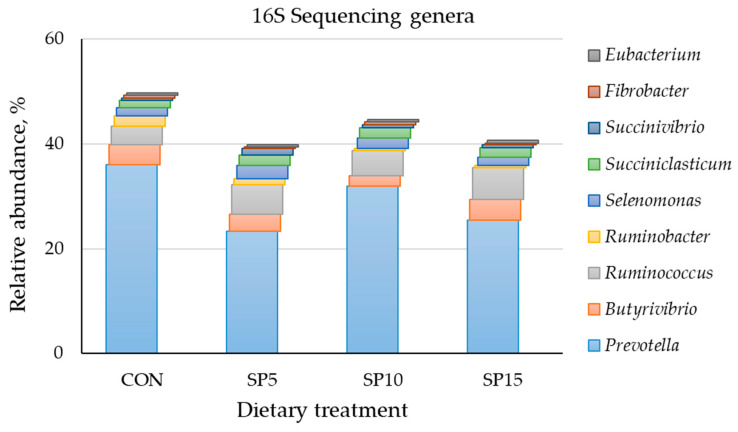
Relative abundance of dominant genera based on the 16S amplicon sequencing in the rumen fluid of the four pooled DNA samples (*n* = 1) representative of the four dietary treatments (CON; SP5; SP10; and SP15) with four different *Spirulina* supplementation levels (0, 5, 10, and 15 g/day, in the concentrate, respectively) at a genus level.

Bacteria of the genus *Prevotella* were the main representatives of the phylum Bacteroidetes in the four dietary treatments (Figure 2). The highest relative abundance was found in the CON (36.0%) compared to the SP5 (23.3%), SP10 (31.9%), and SP15 (25.5%). The predominant bacterial species of this genus were *Prevotella ruminocola* for the four dietary treatments. Moreover, the genus *Butyrivibrio* was the second most dominant for the CON, while the *Ruminococcus*, *Butyrivibrio*, and *Selenomonas* genera were next most abundant for the SP dietary groups (Figure 2).

### 3.2. Relative Abundance of Selected Microorganisms in the Rumen Fluid Samples Using a RT-qPCR Platform

**Figure 3:**
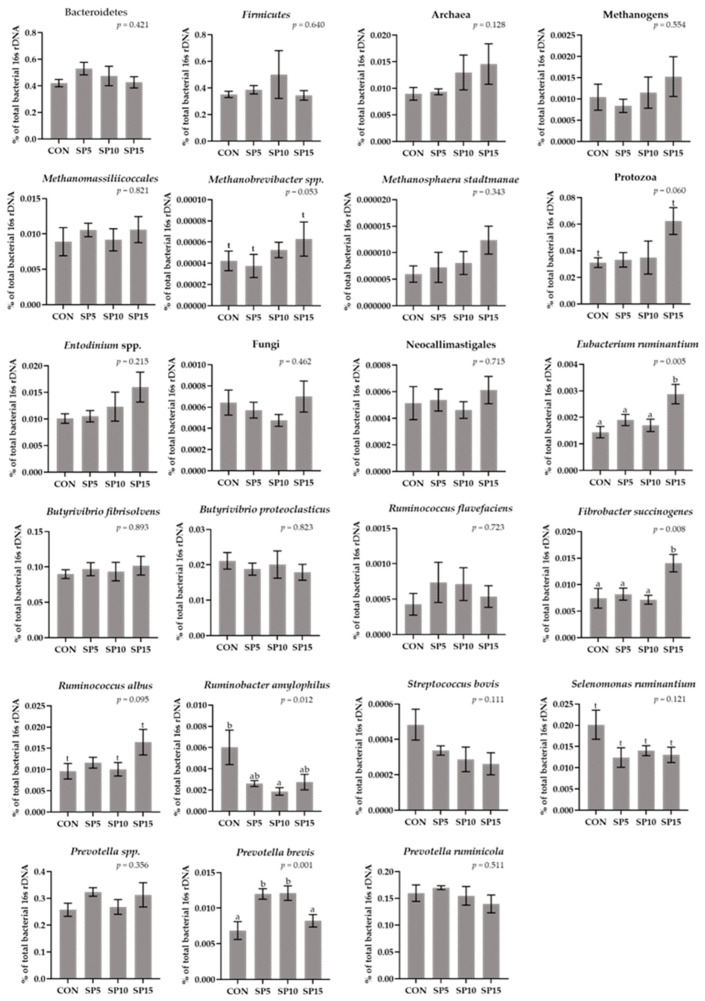
Column bar plots (±Standard error) of the average changes of target microorganisms as a proportion of the total rumen bacterial 16s rDNA in the rumen fluid of ewes-fed diets (CON, SP5, SP10, and SP15) with different levels of *Spirulina* supplementation (0, 5, 10, and 15 g/day, respectively). Bars with different superscript letters (a, b) between dietary treatments differ significantly (*p* < 0.05) and t is referred to values between 0.100 and 0.05. Analysis was conducted using one-way ANOVA and Post hoc analysis was performed using Tukey multiple range test when appropriate.

The relative abundance of Bacteroidetes and Firmicutes as well as the Firmicutes:Bacteroidetes ratio did not significantly differ among the four dietary treatments (Figure 3, Table S5). The relative abundance of Archaea, Fungi, and total Methanogens did not significantly differ in the four dietary treatments, and Protozoa tended to increase in the SP15 (*p* = 0.060) (Figure 3, Table S5). The relative abundance of the species *Prevotela brevis* was significantly increased (*p* = 0.001) in the SP5 and the SP10 compared to the CON and SP15 (Figure 3, Table S5). In addition, *Methanobrevibacter* tended to increase in the SP15 (*p* = 0.053) (Figure 3, Table S5). A significant decrease (*p* = 0.012) was observed in the relative abundance of *Ruminobacter amylophilus* in the SP10 compared to the CON-fed ewes (Figure 3, Table S5). Furthermore, the relative abundance of *Fibrobacter succinogenes* (*p* = 0.008) and *Eubacterium ruminantium* (*p* = 0.005) were significantly increased in the SP15, while the relative abundance of *Ruminococcus albus* tended to increase (*p* = 0.095) in the SP15 compared to the CON and SP5-fed ewes (Figure 3, Table S5).

### 3.3. Relative Abundance of Selected Microorganisms in the Rumen Solid Particle Using RT-qPCR Platform

**Figure 4:**
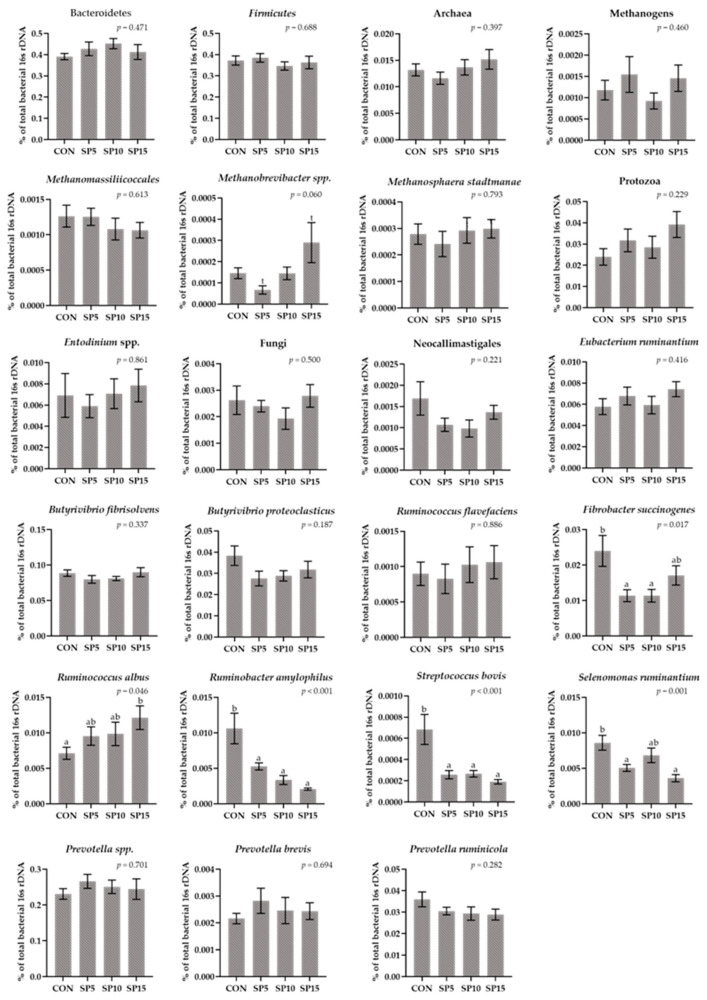
Column bar plots (±Standard error) of the average changes of target microorganisms as a proportion of the total rumen bacterial 16s rDNA in the rumen solid particles of ewes-fed diets (CON, SP5, SP10, and SP15) with different levels of *Spirulina* supplementation (0, 5, 10, and 15 g/day, respectively). Bars with different superscript letters (a, b) between dietary treatments differ significantly (*p* < 0.05) and t is referred to values between 0.100 and 0.05. Analysis was conducted using one-way ANOVA and Post hoc analysis was performed using Tukey multiple range test when appropriate.

The relative abundance of Bacteroidetes, Firmicutes, and the ratio Firmicutes:Bacteroidetes did not significantly differ among the four dietary treatments (Figure 4, Table S6). In addition, the relative abundance of Protozoa, Fungi, Archaea, and total Methanogens did not significantly differ among the dietary treatments, while the relative abundance of *Methanobrevibacter* tended to increase (*p* = 0.060) in the SP15 compared to the SP5-fed ewes (Figure 4, Table S6). The relative abundance of *Ruminobacter amylophilus* and *Streptococcus bovis* were significantly decreased (*p* < 0.001) in all SP-fed ewes (Figure 4, Table S6). Moreover, the relative abundance of *Fibrobacter succinogenes* was significantly reduced (*p* = 0.017) in the SP5 and SP10 compared to the CON, while the relative abundance of *Selenomonas ruminantium* was significantly reduced (*p* = 0.001) in the SP5 and SP15 compared to the CON (Figure 4, Table S6). In contrast, the relative abundance of *Ruminococcus albus* was significantly increased (*p* = 0.046) in the SP15 compared to the CON group (Figure 4, Table S6).

## 4. Discussion

Studies to date have mainly focused on assessing the effect of *Spirulina* supplementation on animal performance and product quality. Our previous study suggested that *Spirulina* dietary supplementation in the three different studied levels did not affect ewe performance, while it improved the oxidative status of both ewes’ organism and milk [6]. Nevertheless, for a more thorough evaluation of *Spirulina* as a feed additive, it was critical not only to assess the effect on animal health status and product quality but also to inspect its impact on feed degradation potential as reflected by rumen inhabitation.

As far as is known, this is the first in vivo study that has investigated the effect of supplementing different levels of *Spirulina* on the rumen microbiota of ewes and more specifically the specific bacterial species that are involved in the feed degradation processes as well as methane formation, in both rumen fluid and solid. For a more thorough approach, the advent of rapid and robust long-read sequencing by Oxford Nanopore was used to comprehensively evaluate the effect of *Spirulina* supplementation on the rumen bacteriome of ewes. Following this initial bacteriome cataloguing, the relative abundance of selective rumen microorganisms was assessed using a well-assessed RT-qPCR platform. By such an approach, previous work from our research group [12,21] unveiled important findings and an in-depth understanding of the rumen microbiota under the influence of feed additives to diets of small ruminants. Discrepancies in the abundance levels of dominant phyla between NGS and qPCR approaches may lie in both different analytical workflows (pool DNA samples on NGS vs. individually on qPCR) and the dissimilar amplification regions and amplicon length. Although these minor inconsistencies were not subversive or significant, this partial incompatibility between the two methodologies remains a controversial topic necessitating further research.

### The Spirulina Supplementation Demonstrated Important Findings in the Modification of the Populations of Rumen Microorganisms

In the past, studies evaluated the microbial-modulating activity of *Spirulina* and its inhibitory activity against Gram-negative and Gram-positive bacteria [26]. *Spirulina* produces extracellular metabolites with antibacterial activity [27,28,29,30]. Conversely, *Spirulina* extracellular products, obtained from an advanced exponential phase culture and separated by filtration, have been reported to significantly promote the growth of lactic acid bacteria in vitro [26]. Regarding the *Spirulina* biomass, although better growth and survival have been linked to the high level of nitrogenous substances, specifically free amino acids [31], phenolic compounds have been acknowledged to exert antimicrobial or bacteriostatic activities and improve probiotic growth [32].

Supplementing *Spirulina* to the diet of ewes did not significantly affect the relative abundance of total methanogens. However, in its highest dietary inclusion level (SP15), the relative abundance of the key methanogenic Archaeon taxon *Methanobrevibacter* tended to increase in both the liquid and solid rumen fractions. Archaea make up about 0.3 to 4% of the rumen microbial DNA (16s and 18s rRNAs) [33]. Almost all the Archaea are the so-called methanogens that have been related to methane (CH_4_) production and the subsequent livestock emissions [34]. In our study, the relative abundance of the total rumen methanogens in both the fluid and solid fraction is in partial contrast to a study that reported that the addition of 10 g *Chlorella vulgaris*/kg (10% ether extract content), increased total methanogens in goats’ rumen fluid [10]. In contrast, different findings were observed by the supplementation of microalgae rich in ether extract (55.6% ether extract content) and polyunsaturated fatty acids (PUFA), especially DHA (22% of total fatty acids), such as *Schizochytrium* spp. [14,15]. Therefore, it appears that the manipulation of rumen methanogens by the administration of microalgae in ruminants’ diet may not be strictly related to their ether extract and fatty acid (e.g., DHA) content but also other compounds and secondary metabolites presented in algae biomass can impair methanogens abundance. Further to the metabolic function of methanogens per se, a mutualistic mode of action involving the *Selenomonas ruminantium* has been reported in in vitro co-cultures [35]. However, in our study, the relative abundance of *Selenomonas ruminantium* was significantly reduced in the solid fraction of the SP5 and SP15.

Furthermore, methanogens coexist also in symbiotic interactions with protozoa [36] and thus, there is a linear relationship between protozoa and CH_4_ emissions as well [37]. Nevertheless, plant extracts have been associated with the manipulation of protozoa, however, thus far, there is no applicable approach to control the rumen protozoa. [38]. The numerical increase in protozoa in the group with the highest inclusion level (SP15), both in rumen fluid and solid, is in agreement with the reported increase in protozoa when *Chlorella vulgaris* (10 g/kg of concentrate dry matter) was included in goat diets [10].

A meta-analysis reported that rumen defaunation (absence of rumen protozoa), can suppress both the abundance and activity of fibrolytic microorganisms, and more specifically of anaerobic fungi, and bacterial species namely *Ruminococcus albus* and *Ruminococcus flavefaciens* and thus impairs the overall rumen organic matter digestibility [37]. The incorporation of 10 g of *Chlorella vulgaris* in goat diets significantly reduced the population of *Ruminococcus albus* in their rumen fluid [10]. Among the former cellulolytic bacteria, *Fibrobacter succinogenes* demonstrates the strongest cellulolytic activity [39] and *Ruminococcus albus* has been considered a cellulolytic specie of major importance [40]. The fact that the highest inclusion level of *Spirulina* in ewe diets resulted in a higher relative abundance of major cellulolytic bacterial species (*Eubacterium ruminantium* and *Fibrobacter succinogenes* in rumen fluid, and *Ruminococcus albus* in the solid fraction) could be considered as an interesting finding which requires further investigation as it was accompanied by an increasing abundance trend in both protozoa and methanogenic species in the rumen of ewes.

Supplementing *Spirulina* as an additive in ewe diets reduced the population of specific bacterial species with prominent amylolytic activity such as *Ruminobacter amylophilus*, *Streptococcus bovis*, and *Selenomonas ruminantium*. Especially regarding the *Ruminobacter amylophilus*, similar results were reported in both the NGS and RT-qPCR methods. Shifting starch degradation from the rumen to the small intestine digestion may improve feed conversion efficiency since starch digestion in the small intestine can provide proportionally more net energy to the host [41,42,43]. The reduced starch fermentation in the rumen is desirable to prevent acidosis and to increase the supply of glycogenic substrates for the host [44]. Highly fermentable starch in the rumen increases the production of volatile fatty acids (VFAs) and especially lactic acid, which can lower the rumen’s pH and affect the population and metabolism of cellulolytic microorganisms, leading to reduced digestibility. These phenomena are more frequent in the high-yield ruminants where high-grain diets are administrated to fulfil their increasing energy and nutrient demands. In these cases, metabolic disorders such as acute and subacute rumen acidosis, galactosemia, haemoconcentration, decarbonization of the rumen mucosa, liver abscess, and polioencephalomalacia can occur [45]. Therefore, our results regarding the effect of *Spirulina* supplementation in the relative abundance of the targeted amylolytic bacterial species may deserve further evaluation by assessing changes in ruminal pH, VFA production, and amylase activity in the rumen fluid. *Streptococcus bovis* has been also associated with lactic acid production which usually occurs in the rumen of animals consuming low forage or high fermentable starch diets. Excess digestible carbohydrates can lead to the overgrowth of starch-metabolizing, acid-resistant, lactic acid-producing bacteria such as *Streptococcus bovis* and *Lactobacillus* spp. [46,47,48]. When fed a high-starch diet, starch-fermenting bacteria like *Streptococcus bovis* prevail and can compete with other ruminal species [48]. Moreover, the risk of subacute ruminal acidosis in cows has been linked with the rate of starch intake and fermentation [42,45,49,50,51]. Besides, ruminal acidosis occurs during abrupt changes from a roughage-based diet to a low-cellulose diet with a high carbohydrate content [46]. A review evaluated the microbial-modulating activity of *Spirulina* that may prevent dysbiosis, benefit probiotic-species growth bacteria [19].

## 5. Conclusions

The inclusion of *Spirulina* in the diets of dairy ewes slightly modulated selected rumen microbes majorly regulating feed degradation with the most profound changes revealed for the higher supplementation level (15 g of *Spirulina*/ewe/day) suggesting a dose-dependent effect. Although selected cellulolytic bacteria expanded their niche against amylolytic microbes belonging to the genera *Ruminobacter* and *Streptococcus*, an unfavourable trend for an increase of *Methanobrevibacter* was observed. The former requires special attention as future dietary strategies should holistically consider the livestock environmental burden as well. More research and validation approaches at functional level are necessary to further explore the effect of *Spirulina* on rumen biochemistry.

## Figures and Tables

**Table 1:** Relative abundance (%) in the metagenomic sequencing of the genomic DNA of the rumen fluid samples (*n* = 1; pooled group samples) from the four dietary treatments (CON, SP5, SP10, and SP15).

Dietary Treatments	Phyla (%)			
	Bacteroidetes	Firmicutes	Proteobacteria	Firmicutes:Bacteroidetes
CON	39.3	50.7	7.9	1.29
SP5	26.5	59.4	12.5	2.24
SP10	36.1	55.3	6.2	1.53
SP15	29.2	63.0	5.8	2.16

CON: Control dietary treatment with no *Spirulina* supplementation. SP5: Dietary treatment with 5 g *Spirulina*/day included in the concentrate. SP10: Dietary treatment with 10 g *Spirulina*/day included in the concentrate. SP15: Dietary treatment with 15 g *Spirulina*/day included in the concentrate.

## Data Availability

All data are available within the manuscript and Supplementary Materials.

## Supplementary Materials

The following supporting information can be downloaded at. https://www.mdpi.com/article/10.3390/ani13040740/s1. Table S1: Concentrate ingredients of the four dietary treatments (CON, SP5, SP10, SP15) with different levels of *Spirulina* (0, 5, 10, and 15 g/day); Table S2: Chemical composition (g/kg DM), and fatty acids (g/100 g total fatty acids) of the forages (alfalfa hay and wheat straw), the concentrate, and *Spirulina* (SP). Table S3: Average daily feed intake (g/ewe/day) and nutrients intake (g/ewe/day) for the four dietary treatments (CON, SP5, SP10, SP15) with different levels of *Spirulina* supplementation (5, 10, 15 g/ewe/day); Table S4: Sequences of primers used for RT-PCRs, genomic regions of PCR amplification, primer efficiency, amplicon size, and hybridization temperature; Table S5: Relative abundance of the microorganisms in ewes’ rumen fluid for the four dietary treatments (CON, SP5, SP10, SP15) with different levels of *Spirulina* (0, 5, 10, and 15 g/day); Table S6: Relative abundance of the microorganisms in ewes’ rumen solid for the four dietary treatments (CON, SP5, SP10, SP15) with different levels of Spirulina (0, 5, 10, and 15 g/day).
