# Supplementary material for: Effect of Spirulina Dietary Supplementation in Modifying the Rumen Microbiota of Ewes
Source: Animals (Basel). 2023 Feb 19;13(4):740. doi: 10.3390/ani13040740 (PMC9952741; doi:10.3390/ani13040740)
Supplement: Supplementary file 1 [file animals-13-00740-s001.zip › animals-2201570-supplementary.pdf]

Table S1. Concentrate ingredients of the four dietary treatments (CON, SP5, SP10, SP15) with different levels of *Spirulina* (0, 5, 10, and 15 g/day).

	Concentrates			
	CON	SP5	SP10	SP15
Spirulina (g/ewe/day)	0	5	10	15
Ingredients (g/kg)				
Maize grain	344	344	344	344
Barley	200	200	200	200
Wheat middlings	100	100	100	100
Sunflower meal	160	160	160	160
Soybean meal	155	155	155	155
Premix mineral and vitamins	41	41	41	41

CON: control dietary treatment
SP5: dietary treatment with 5 g *Spirulina*/ewe/day
SP10: dietary treatment with 10 g *Spirulina*/ewe/day
SP15: dietary treatment with 15 g *Spirulina*/ewe/day

Table S2. Chemical composition (g/kg DM), and fatty acids (g/100 g total fatty acids) of the forages (alfalfa hay and wheat straw), the concentrate, and *Spirulina* (SP).

Chemical composition (g/kg DM)				
	Alfalfa hay	Wheat straw	Concentrate	*Spirulina*
Dry matter	894	928	902.4	931.5
Ash	93	76	8.6	164.3
Crude protein	200	48	200.3	571.0
Ether extract	28	16	23.6	4.2
Ash free neutral detergent fiber	366	728	153	224
Acid detergent fiber	325	493	52.9	40
Main fatty acids (g/100 g total fatty acids)	Alfalfa hay	Wheat straw	Concentrate	*Spirulina*
C14:0	2.35	6.16	0.18	0.49
C16:0	43.53	33.38	0.22	36.00
C16:1 n-7	2.99	-	0.21	10.06
C18:0	6.79	4.28	2.94	8.76
cis-9 C18:1	3.01	9.00	20.83	0.57
cis C18:2 n-6	16.34	26.74	54.14	18.34
C18:3 n-6	-	-	0.01	20.97
C20:0	0.70	1.12	-	-
C18:3 n-3	18.59	11.22	-	-
C20:1 n-9	-	-	3.41	-
C20:2 n-6	-	-	0.59	-
C20:3 n-6	-	-	0.40	0.68
C22:0	1.48	3.99	-	-
C24:0	2.71	1.94	0.27	-

Table S3. Average daily feed intake (g/ewe/day) and nutrients intake (g/ewe/day) of the four dietary treatments (CON, SP5, SP10, SP15) with different levels of *Spirulina* supplementation (5, 10, 15 g/ewe/day).

Average daily feed intake (g/ewe/d)	Dietary treatments (D)			
	CON	SP5	SP10	SP15
Wheat Straw	200	200	200	200

Alfalfa Hay	1000	1000	1000	1000
Concentrate	1500	1500	1500	1500
Nutrients intake (g/day/ewe)	Dietary treatments (D)			
	CON	SP5	SP10	SP15
Dry matter	2433.2	2437.7	2442.2	2446.7
Ash	238.4	239.2	240.0	240.9
Crude protein	510.1	512.9	515.8	518.6
Ether extract	38.5	38.5	38.6	38.6
Ash free neutral detergent fiber	741.0	742.1	743.2	744.3
Acid detergent fiber	503.0	503.2	503.4	503.6

CON: control dietary treatment
SP5: dietary treatment with 5 g *Spirulina*/ewe/day
SP10: dietary treatment with 10 g *Spirulina*/ewe/day
SP15: dietary treatment with 15 g *Spirulina*/ewe/day

Table S4. Sequences of primers used for RT-PCRs, genomic regions of PCR amplification, primer efficiency, amplicon size, and hybridization temperature.

Target Species	Genomic Region of PCR Amplification	Primer Sequencing	Primer Efficiency %	Slope	Amplicon bp	Tm °C
Total bacteria	16s rRNA	F: 5′-CGGCAACGAGCGCAACCC-3′<br>R: 5′-CCATTGTAGCACGTGTGTAGCC-3′	98	−3.378	130	60
Bacteroidetes	16s rRNA	F: 5′-GGARCATGTGGTTTAATTCGATGAT-3′<br>R: 5′-AGCTGACGACAACCATGCAG-3′	98	−3.36	126	62
Firmicutes	16s rRNA	F: 5′-GGAGYATGTGGTTTAATTCGAAGCA-3′<br>R: 5′-AGCTGACGACAACCATGCAC-3′	97	−3.39	126	62
Archaea	16s rRNA	F: 5′-GAGGAAGGAGTGGACGACGGTA-3′<br>R: 5′-ACGGGCGGTGTGTGCAAG-3′	96	−3.43	233	60
Protozoa	18s rRNA	F: 5′-GCTTTCGWTGGTAGTGTATT-3′<br>R: 5′-CTTGCCCTCYAATCGTWCT-3′	97	−3.39	223	55
Entodinium	18s rRNA	F: 5′-GAGCTAATACATGCTAAGGC-3′<br>R: 5′-CCCTCACTACAATCGAGATTTAAGG-3′	97	−3.39	317	59
Total fungi	18s rRNA<br>ITS1	F: 5′-<br>GAGGAAGTAAAAGTCGTAACAAGGTTTC-3′<br>R: 5′-CAAATTCACAAAGGGTAGGATGATT-3′	98	−3.38	120	58
Neocallimastigales	18s rRNA<br>ITS1	F: 5′-TTGACAATGGATCTCTTGGTTCTC-3′<br>R: 5′-GTGCAATATGCGTTCGAAGATT-3′	96	−3.43	110	63
Methanogen	mcrA	F: 5′-TTCGGTGGATCDCARAGRGC-3′<br>R: 5′-GBARGTCGWAWCCGTAGAATCC-3′	95	−3.44	140	58
Methanomassiliicoccales	16s rRNA	F: 5′-TTCTGGGGTAGGGGTAAAATC-3′<br>R: 5′-GTCTGCAGCGTTTACACCCT-3′	97	−3.40	149	62
Methanobrevibacter spp.	16s rRNA	F: 5′-TGGGAATTGCTGGWGATACTRTT-3′<br>R: 5′-GGAGCRGCTCAAAGCCA-3′	95	−3.46	231	60
Methanosphaera stadtmanae	16s rRNA	F: 5′-CTTAACTATAAGAATTGCTGGAG-3′<br>R:5′-TTCGTTACTCACCGTCAAGATC-3′	97	−3.39	150	58
Butyrivibrio fibrisolvens	16s rRNA	F: 5′-TAACATGAGAGTTTGATCCTGGCTC-3′<br>R: 5′-CGTTACTCACCCGTCCGC-3′	97	−3.39	136	58
Butyrivibrio proteoclasticus	16s rRNA	F: 5′-TCCGGTGGTATGAGATGGGC-3′<br>R: 5′-GTCGCTGCATCAGAGTTTCCT-3′	98	−3.38	185	60
Eubacterium ruminantium	16s rRNA	F: 5′-CTCCCGAGACTGAGGAAGCTTG-3′<br>R: 5′-GTCCATCTCACACCACCGGA-3′	98	−3.36	184	62
Ruminococcus flavefaciens	16s rRNA	F: 5′-<br>CGAACGGAGATAATTTGAGTTTACTTAGG-3′	95	−3.42	132	60

		R: 5′-CGGTCTCTGTATGTTATGAGGTATTACC-3′				
Fibrobacter succinogenes	16s rRNA	F: 5′-GCGGGATTGAATGTACCTTGAGA-3′<br>R: 5′-TCCGCCTGCCCCTGAACTATC-3′	98	−3.39	204	60
Ruminococcus albus	16s rRNA	F: 5′-CCCTAAAAGCAGTCTTAGTTCG-3′<br>R: 5′-CCTCCTTGCGGTTAGAACA-3′	98	−3.38	175	62
Ruminobacter amylophilus	16s rRNA	F: 5′-ATGCAAGTCGAACGGTAACAGCAGG-3′<br>R: 5′-GCACCCGTTTCCAGGTGTTGTCC-3′	96	−3.42	115	65
Streptococcus bovis	16s rRNA	F: 5′-TTCCTAGAGATAGGAAGTTTCTTCGG-3′<br>R: 5′-ATGATGGCAACTAACAATAGGGGT-3′	96	−3.43	127	57
Selenomonas ruminantium	16s rRNA	F: 5′-CAATAAGCATTCCGCCTGGG-3′<br>R: 5′-TTCACTCAATGTCAAGCCCTGG-3′	99	−3.35	138	57
Prevotella sp.	16s rRNA	F: 5′-GGTTCTGAGAGGAAGGTCCCC-3′<br>R: 5′-TCCTGCACGCTACTTGGCTG-3′	96	−3.42	121	60
Prevotella brevis	16s rRNA	F: 5′-GGTTTCCTTGAGTGTATTCGACGTC-3′<br>R:5′-CTTTCGCTTGGCCGCTG-3′	98	−3.38	219	64
Prevotella ruminicola	16s rRNA	F: 5′-GAAAGTCGGATTAATGCTCTATGTTG-3′<br>R:5′-CATCCTATAGCGGTAAACCTTTGG-3′	97	−3.39	74	63

Table S5. Relative abundance of the microorganisms in ewes' rumen fluid of the four dietary treatments (CON, SP5, SP10, SP15) with different levels of *Spirulina* (0, 5, 10, and 15 g/day).

	Dietary treatments					
	CON	SP5	SP10	SP15	SEM[a]	*P*
Bacteroidetes	0.422	0.530	0.474	0.427	0.026	0.421
Firmicutes	0.349	0.386	0.500	0.344	0.047	0.640
Firmicutes:Bacteroidetes	0.842	0.778	0.938	0.853	0.054	0.786
Prevotella sp.	0.259	0.324	0.268	0.313	0.015	0.356
Prevotella ruminicola	0.162	0.170	0.155	0.140	0.007	0.511
Prevotella brevis	0.007^{b}	0.012^{a}	0.012^{a}	0.008^{b}	0.001	0.001
Protozoa	0.032^{t}	0.033^{t}	0.035^{t}	0.062^{t}	0.005	0.060
Total Fungi	0.0006	0.0006	0.0005	0.0007	0.0001	0.462
Neocallimastigales	0.0005	0.0005	0.0005	0.0006	0.0001	0.715
Entodinium	0.010	0.010	0.012	0.016	0.001	0.215
Archaea	0.009	0.009	0.013	0.017	0.001	0.128
Total Methanogen	0.001	0.001	0.001	0.001	0.0002	0.554
Methanomassiliicoccales	0.009	0.011	0.009	0.011	0.001	0.821
Methanobrevibacter	0.00001^{t}	0.00001^{t}	0.00006	0.00008^{t}	<0.0001	0.053
Methanosphaera stadtmanae	<0.0001	<0.0001	<0.0001	<0.0001	<0.0001	0.343
Ruminococcus flavefaciens	0.0004	0.0007	0.0007	0.0005	0.0001	0.723
Ruminobacter amylophilus	0.006^{a}	0.003^{ab}	0.002^{b}	0.003^{ab}	0.001	0.012
Ruminococcus albus	0.010^{t}	0.012	0.010^{t}	0.016^{t}	0.001	0.095
Butyrivibrio fibrisolvens	0.090	0.097	0.093	0.103	0.005	0.893
Butyrivibrio proteoclasticus	0.020	0.019	0.020	0.018	0.001	0.823
Fibrobacter succinogenes	0.007^{b}	0.008^{b}	0.007^{b}	0.014^{a}	0.001	0.008
Selenomonas ruminantium	0.020	0.012	0.014	0.013	0.001	0.121
Streptococcus bovis	0.0005	0.0003	0.0003	0.0003	<0.0001	0.111
Eubacterium ruminantium	0.0013^{b}	0.0019^{b}	0.0017^{b}	0.0029^{a}	0.0002	0.005

CON: control dietary treatment
SP5: dietary treatment with 5 g *Spirulina*/ewe/day
SP10: dietary treatment with 10 g *Spirulina*/ewe/day
SP15: dietary treatment with 15 g *Spirulina*/ewe/day
Different superscript letters (a. b) between dietary treatments differ significantly ($P < 0.05$) and t is referred to values between 0.05 and 0.100 ($0.05 < t < 0.10$)

[a] SEM = standard error mean
P = *P* value

Table S6. Relative abundance of the microorganisms in ewes' rumen solid of the four dietary treatments (CON, SP5, SP10, SP15) with different levels of *Spirulina* (0, 5, 10, and 15 g/day).

	Dietary treatments					
	CON	SP5	SP10	SP15	SEM[a]	*P*
Bacteroidetes	0.390	0.428	0.452	0.413	0.014	0.472
Firmicutes	0.952	0.900	0.768	0.880	0.011	0.688
Firmicutes:Bacteroidetes	0.960	0.921	0.789	0.906	0.034	0.313
Prevotella spp.	0.230	0.266	0.251	0.244	0.010	0.701
Prevotella ruminicola	0.035	0.031	0.029	0.029	0.001	0.282
Prevotella brevis	0.023	0.028	0.025	0.024	0.0002	0.694
Protozoa	0.026	0.032	0.029	0.039	0.003	0.229
Total Fungi	0.003	0.002	0.002	0.003	0.0002	0.500
Neocallimastigales	0.018	0.011	0.010	0.014	0.0001	0.221
Entodinium	0.070	0.059	0.071	0.079	0.001	0.861
Archaea	0.013	0.012	0.014	0.015	0.001	0.397
Total Methanogen	0.0013	0.0016	0.009	0.0015	0.0001	0.460
Methanomassiliicoccales	0.0013	0.0013	0.0011	0.0011	0.0001	0.613
Methanobrevibacter	0.00018	0.00007^{t}	0.00014	0.00029^{t}	<0.0001	0.060
Methanosphaera stadtmanae	0.0003	0.0002	0.0003	0.0003	<0.0001	0.793
Ruminococcus flavefaciens	0.0008	0.0008	0.0010	0.0011	0.0001	0.886
Ruminobacter amylophilus	0.012^{a}	0.006^{b}	0.004^{b}	0.002^{b}	0.001	<0.001
Ruminococcus albus	0.007^{b}	0.010^{ab}	0.010^{ab}	0.012^{a}	0.001	0.046
Butyrivibrio fibrisolvens	0.087	0.080	0.081	0.090	0.002	0.377
Butyrivibrio proteoclasticus	0.037	0.028	0.029	0.032	0.002	0.187
Fibrobacter succinogenes	0.023^{a}	0.010^{b}	0.009^{b}	0.017^{ab}	0.002	0.017
Selenomonas ruminantium	0.010^{a}	0.005^{b}	0.007^{ab}	0.004^{b}	0.001	0.001
Streptococcus bovis	0.0007^{a}	0.0003^{b}	0.0003^{b}	0.0002^{b}	0.0001	<0.001
Eubacterium ruminantium	0.006	0.007	0.006	0.007	0.0004	0.416

CON: control dietary treatment
SP5: dietary treatment with 5 g *Spirulina*/ewe/day
SP10: dietary treatment with 10 g *Spirulina*/ewe/day
SP15: dietary treatment with 15 g *Spirulina*/ewe/day
Different superscript letters (a. b) between dietary treatments differ significantly ($P < 0.05$) and t is referred to values between 0.05 and 0.100 ($0.05 < t < 0.10$)
[a] SEM = standard error mean
P = *P* value
